# Remyelinating Drugs at a Crossroad: How to Improve Clinical Efficacy and Drug Screenings

**DOI:** 10.3390/cells13161326

**Published:** 2024-08-08

**Authors:** Aland Ibrahim Ahmed Al Jaf, Simone Peria, Tommaso Fabiano, Antonella Ragnini-Wilson

**Affiliations:** 1 Department of Biology, University of Rome “Tor Vergata”, Via della Ricerca Scientifica 1, 00133 Rome, Italy; 2 Department of Life Sciences, University of Trieste, 34127 Trieste, Italy

**Keywords:** myelin, central nervous system, oligodendrocytes, multiple sclerosis, neurodegeneration, remyelination, drug discovery, artificial Intelligence, iPSCs, organoids

## Abstract

Axons wrapped around the myelin sheath enable fast transmission of neuronal signals in the Central Nervous System (CNS). Unfortunately, myelin can be damaged by injury, viral infection, and inflammatory and neurodegenerative diseases. Remyelination is a spontaneous process that can restore nerve conductivity and thus movement and cognition after a demyelination event. Cumulative evidence indicates that remyelination can be pharmacologically stimulated, either by targeting natural inhibitors of Oligodendrocyte Precursor Cells (OPCs) differentiation or by reactivating quiescent Neural Stem Cells (qNSCs) proliferation and differentiation in myelinating Oligodendrocytes (OLs). Although promising results were obtained in animal models for demyelination diseases, none of the compounds identified have passed all the clinical stages. The significant number of patients who could benefit from remyelination therapies reinforces the urgent need to reassess drug selection approaches and develop strategies that effectively promote remyelination. Integrating Artificial Intelligence (AI)-driven technologies with patient-derived cell-based assays and organoid models is expected to lead to novel strategies and drug screening pipelines to achieve this goal. In this review, we explore the current literature on these technologies and their potential to enhance the identification of more effective drugs for clinical use in CNS remyelination therapies.

## 1. Introduction

The finding that cognitive and neuromuscular functions could be restored in animal models of demyelination disease through the stimulation of specific neural and/or glial stem cell precursors has reinforced the hope of developing therapeutic strategies for several neurodegenerative diseases of the Central Nervous System (CNS) characterized by primary or secondary demyelination, such as Multiple Sclerosis (MS), Neuro-Myelitis Optica (NMO), and Acute Disseminated Encephalomyelitis (ADE) [1,2]. Additionally, recent studies on the mechanism by which age-related cognitive dysfunctions such as Alzheimer’s and Parkinson’s emerge and develop suggested that these conditions might also benefit from remyelination therapies [3].

Among the demyelinating diseases, CNS remyelination in MS was studied in detail due to the potential benefits that remyelination therapies could offer in combination with Disease-Modifying Therapies (DMTs). MS is an autoimmune inflammatory demyelination pathology of the CNS that affects more than 2.8 million people worldwide. Often, it occurs in the early stages of working and family life, with high personal, familial, and social costs. MS generally causes various debilitating symptoms such as ataxia, sensory impairment, cognitive dysfunction, and fatigue that often prevent people from continuing to work. MS has different characteristics, depending on the progression of the disease: Relapsing–Remitting MS (RRMS), Primary Progressive MS (PPMS), Secondary Progressive MS (SPMS), and Clinically Isolated Syndrome (CIS), are the main recognized forms. Patients with RRMS are characterized by autoimmune attacks against the myelin of the CNS that cause neuroinflammation and, therefore, motor and functional deficits. These phases are followed by relapses, resulting in functional recovery of neurological symptoms [4,5].

Remyelination therapies are expected to substantially improve MS patients’ quality of life by retarding the disease progression, protecting axons from neurodegeneration and restoring lost functions [4]. Unfortunately, there is still a lack of fundamental understanding of how to promote remyelination in the adult human brain nor has it been clarified why MS patients progress differently in the secondary phases [2,4,5]. Studies of the mechanism by which remission occurs in RRMS highlighted a previously underappreciated reparatory activity of the adult brain: myelin can regenerate in demyelinating lesions [1,6]. Unfortunately, after several years, most patients with RRMS develop SPMS, whereas neurological functions decrease over time independently of relapse activity. Treatment with DMTs is available to modulate the immune system in MS patients. The most challenging aspect of the SPMS form is the lack of an efficient regenerative response to remyelination. About 10–15% of MS patients do not have relapses, and the disease progresses from the beginning with a gradual increase in neurological symptoms (PPMS). Often, a spastic gait disorder develops over time and more rarely a progressive cerebellar syndrome [7]. The diagnosis of MS is complex, emphasizing the importance of early management to control disability and slow disease progression, thereby delaying the onset of the secondary progressive phase.

In the past ten years, several pharmacological approaches to remyelination have been investigated. Among them, new and repurposed drugs can be included [2,8]. Despite the primary targets being different, recent studies indicate that several candidates enhance the maturation of Oligodendrocyte Precursor Cells (OPCs), independently of their primary targets [3]. The larger group of promyelinating drugs identified led to the accumulation of 8,9-unsaturated sterols by inhibiting a narrow set of enzymes of the cholesterol biosynthetic pathway: namely Emopamil Binding Protein (EBP), Sterol 14-demethylase (CYP51), and Sterol C-14 reductase (S14R) [3,9,10]. A smaller set of remyelination drugs does not impact cholesterol biosynthesis nor cause accumulation of sterol precursors, although they share the ability to bind to the Smoothened (Smo) receptor of the Hedgehog (Hh) pathway (Table 1) [10,11,12]. How Smo favours remyelination is still poorly understood [13].

Despite the advances, none of the hit compounds of either class has successfully passed the final clinical evaluation for MS pathology yet (Table 2) [4]. Therefore, it is necessary to rethink drug selection strategies and/or patient stratification.

Aside from MS pathology, remyelination therapies are expected to have a wider application to diseases affecting myelin in which glial cell differentiation is affected. Artificial Intelligence (AI)-driven clinical reports, drug screening, and results analysis, combined with the development of faster methods to derive OPCs from induced Pluripotent Stem Cells (iPSCs) isolated from patient samples are expected to provide new impetus to future drug screening campaigns [37].

In this review, we present the current state of knowledge regarding the mechanisms by which Neural Precursor Cells (NPCs) mature into myelinating cells under both healthy and MS conditions. We also explore the processes of remyelination in these contexts and examine the latest technologies aimed at improving the attrition rate of CNS promyelinating drugs.

## 2. The Biological Basis of Remyelination in Healthy and Multiple Sclerosis Pathological Conditions

In humans, most regions of the brain and spinal cord are fully myelinated by the third decade of life [38]. Upon CNS myelin injury, remyelination occurs spontaneously in healthy individuals and allows restoration of neuronal functionality after myelin damage [1,39,40,41].

### 2.1. Cells Participating in Remyelination

In the adult mammalian brain, different neural stem cell niches can be identified. Neural Stem Cells (NSCs) reside primarily in niches in the Subventricular Zone (SVZ) of the lateral ventricle, located along the layer of ependymal cells, and the Sub Granular Zone (SGZ) of the Dentate Gyrus (DG) of the hippocampus. The NSCs in the SVZ generate interneurons of the olfactory bulb and Corpus Callosum (CC) oligodendrocytes; the SGZ develops neurons and astrocytes [42,43]. In the stem cell niches of the SVZ, the NSCs show heterogeneity: quiescent Neural Stem Cells (qNSCs) stay in a resting phase and start dividing again when they receive signals to become active Neural Stem Cells (aNSCs). Genetic and fate mapping studies performed in murine models indicate that the pool of qNSCs represents a reservoir ready to be recruited to stimulate neurogenesis and oligodendrogenesis: they divide slowly, are highly resistant to stress, can survive antimitotic drugs or irradiation, and regenerate in the SVZ [44,45,46].

There is limited knowledge about the gene regulatory networks that maintain the pool of qNSCs and aNSCs [47]. Extracellular ligands play a crucial role in regulating quiescent and active NSCs balance by facilitating self-renewal, differentiation, cell adhesion, and migration. Several extracellular factors involved in cell specification and signalling include basic Fibroblast Growth Factor (bFGF), Epidermal Growth Factor (EGF), Stromal cell-derived factor 1 (SDF-1), Sonic Hedgehog (Shh), Bone Morphogenetic Proteins (BMPs), and Neurogenic locus notch homolog protein (Notch) [42].

In adult mammalian CNS, parenchymal OPCs (pOPCs) also participate as the second wave in remyelination, after local myelinating Oligodendrocytes (OLs) reactivation [48,49,50,51]. In the adult mammalian prefrontal cortex, pOPCs respond quickly to acute demyelination, expanding the lesion within seven days and producing OLs two weeks after the lesion as NSCs-generated cells expressing Neural antigen 2 (*NG2*) do not increase significantly at lesions until four weeks after demyelination and produce fewer OLs than pOPCs [52,53,54]. Using Cuprizone-induced demyelination of the CC showed that pOPCs compete with NPCs-derived OPCs at lesion indicating that local pOPCs can function as primary stem cell types that respond to remyelination signals and for acute repair of demyelination lesions [55,56]. However, they can only respond to local injury by lacking the ability to migrate large distances during remyelination [55]. In murine and zebrafish models, the remyelination by pre-existing OLs is limited compared to newly formed OLs [48,50]. Since similar results were obtained by studying post-mortem MS tissues and animal models, it is generally believed that NSCs present in the SVZ are stimulated as a third cell population capable of remyelination [57,58,59,60,61,62,63], and that pOPCs and NSCs-derived OPCs may compete in time and space with their relative contributions being dictated by the relative abundance, recruitment, and rapidity of repair by the local remyelinating cell populations [55].

### 2.2. Signals Promoting Neural Precursor Cell Reactivation and Oligodendrocyte Precursor Cell Differentiation in the Adult Brain

NSCs secrete various soluble factors, such as growth and neurotrophic factors or cytokines, to protect existing neural cells and replace lost ones. An increase in bFGF and Retinoic Acid (RA) leads to the differentiation of NSCs into neurons. EGF, Ciliary Neurotrophic Factor (CNTF), and BMPs enhance their differentiation into astrocytes. The upregulation of Platelet-Derived Growth Factor (*PDGF*) strengthens their development into oligodendrocytes [42].

Which signals promote NSCs to the OPCs lineage was the subject of several studies [46,64]. The temporal activation of the morphogen Shh is critical for the proliferation and increased generation of OPCs from NPCs [46,52,65]. The exogenous delivery of Shh to the brain lesion of Cuprizone mice results in a degree of attenuation of the lesion with increased OPC survival, proliferation, and differentiation [52,54,64]. *Shh* is expressed in the demyelinated area of the CC by a subset of pOPCs that contribute to remyelination [52,64,66].

Shh signalling is received by the Patched (Ptch) receptor. Ptch binds the Shh ligand and transduces a signal to the Smo receptor. Smo is a seven-pass transmembrane receptor that belongs to the Frizzled family of G protein-coupled receptors (GPCRs) that, depending on the ligand and cell type, through the Glioma-associated oncogene family zinc finger proteins (Gli1-3) regulates several intracellular pathways besides oligodendrogenesis in adult stem cells [67,68,69,70]. How Smo can be differently regulated by different ligands is an active field of study [8,52,53,54,64,67,68,69,70,71]. A recent study shows that genetic activation of *Smo* by Shh significantly increased numbers of OPCs while decreasing oligodendrocyte differentiation, as examined in the mice CC during development and after Cuprizone demyelination and remyelination. However, upon loss of function with the conditional ablation of *Smo*, myelination in the same scenarios is unchanged, indicating that OPCs might regulate their proliferation or differentiation state through Smo [71].

Smo activation results in *Gli1* gene expression [54,72]. Temporal and partial inhibition of *Gli1* in NSCs results in their early maturation in OPCs [73]. These data agree with the view that inhibition of Smo/Gli1 signalling favours NPCs to OPCs and their maturation in OLs. The observation that a pharmacological blockade of the Smo/Gli1 signal transduction pathway using GSA-10 improves OPC recruitment at the CC in Lysolecithin-treated Glast-CreERT2-YFP reporter mice and stimulates the ability of the Oli-neuM oligodendroglia cell line to mature in OLs that remyelinate synthetic axons in vitro supports the view that Smo/Gli1 signalling inhibition favours OPC differentiation in OLs [11,23].

Stepwise differentiation of OPCs into mature OLs requires tight and timely regulated expression of structural and myelin-associated regulatory genes [38,65,74]. Genetic fate-mapping studies showed that pre-OPCs can be defined by the expression of PDGF Receptor alpha (*PDGFRα*) and the *NG2* proteoglycan [75]. Pre-OPCs express higher levels of G protein-coupled receptor 17 (*GPR17*) and Brain-enriched myelin-associated protein 1 (*Bcas1*) and low levels of Ectonucleotide pyrophosphatase/phosphodiesterase 8 (*Enpp8*) and *BMP4* compared to OPCs. Before axon engagement, OLs express low *GPR17* and higher levels of *Enpp8* and Myelin Regulatory Factor (*MyRF*) [76]. Mature oligodendrocytes sequentially express myelin genes: 2′,3′-Cyclic-nucleotide 3′-phosphodiesterase (*CNPase*), Myelin Basic Protein (*MBP*), myelin Proteolipid Protein 1 (*PLP1*), Myelin-Associated Glycoprotein (*MAG*) and, just before axon engagement, Myelin and lymphocyte protein (*Mal*) and Oligodendrocytic myelin paranodal and inner loop protein (*Opalin*) [38,74]. Once matured into myelinating cells, oligodendrocytes extend their processes over the demyelinated axons and wrap them in multiple layers. Remyelinated axons can be morphologically identified as they have a thinner myelin sheath and shortened internodal lengths [77,78].

### 2.3. Basis of Remyelination Failure in Multiple Sclerosis

Post-mortem histopathology analysis of the brains of MS patients shows that remyelination can occur widely in the CNS of RRMS patients, but disease progression makes myelin turnover insufficient to compensate for demyelinating damage [4,79]. Interestingly, a subpopulation of human-derived OPCs (OLIG6+, Opalin+), specifically involved in remyelination, is underrepresented in MS patients, as determined by single-cell transcriptomic analyses of the human stem cell-derived oligodendrocyte lineage [80,81,82]. Cumulative evidence indicates that the inflammatory environment formed by activated microglia, astrocytes, and the immune system interferes with oligodendrogenesis [82,83,84]. These observations suggest that a specific subpopulation of OPCs cannot mature during MS pathology, possibly because of the inflammatory environment to which they are exposed or to a higher sensitivity of MS-OPCs to hypoxia and apoptosis [38,80,82]. Understanding how inflammation affects OPC differentiation and/or the genetic cues that regulate this process will help in the development of novel strategies for the selection of remyelination agents [37,85,86].

The integrity of the Blood–Brain Barrier (BBB) is chronically impaired in MS pathology. Consequently, activated peripheral immune cells cross into the CNS through the damaged BBB, ultimately leading to immune-mediated tissue injury. Fibrinogen is histopathologically associated with developing demyelination and inflammation and is observed at the perivascular edges of MS lesions, suggesting that blood proteins leak into the CNS and maintain chronic inflammation. Peripheral immune cells crosstalk with glial cells by secreting various cytokines, influencing each other [87]. Inflammatory infiltrates of active MS lesions consist mainly of monocyte-derived macrophages and proliferating microglia with a small contribution of CD8+ T and CD4+ T cells and B cells [83,88,89]. In early MS lesions, myeloid cells participate in the removal of myelin debris [90,91]. The most acute lesions comprise myeloid cells filled with lysosomal inclusion of myelin components, such as MBP, MAG, and CNPase [92]. In later stages, MAG and CNPase are gradually depleted, while major myelin proteins such as MBP remain within phagocytes, which begin to show signs of cytoplasmic lipid depositions for Oil red O and hypertrophic astrocytes that contain an enlarged cytoplasm and often multiple nuclei. Immune cells secrete neurotoxic products, including Reactive Oxygen Species (ROS), glutamate, cytokines, and chemokines. Additionally, they induce an immune response, altering the cellular metabolism of neurons and their axons. This inflammatory response is believed to be an attempt to restore homeostasis and prevent further damage. However, in the long term, they cause intrinsic stress in the CNS, disrupt homeostasis, and ultimately lead to neurodegenerative diseases [87,93]. Poor regeneration and loss of functional recovery occur when the insufficient clearance or maturation of OPCs in lesions leads to persistent scars. Molecules within the scar, such as chondroitin sulfate proteoglycans, semaphorins, and ephrins, have inhibitory properties on axon growth [83,94].

### 2.4. Pharmacological Approaches for Remyelination

Most of the pharmacological approaches to CNS remyelination are based on the observation that OPCs accumulate in an undifferentiated state at lesions and fail to express myelin genes [8,25]. Due to the difficulty in culturing primary OPCs, early phenotypical drug library screens were performed in mouse oligodendroglia cell lines. Using the oligodendroglia cell line Oli-neu, Joubert and colleagues (2010) identified RA and cell cycle inhibitors as main hits, using as an endpoint the increase in the O4 marker [95]. A2B5+/O4− defines the most immature oligodendrocyte precursors, while O4+/O1− defines intermediate precursors (pre-OLs), and O1+ defines oligodendrocytes at a more mature stage. Following the demonstration that *MyRF* transcription factor expression allows OPCs to enter the pre-myelination stage [76], we exploited the Oli-neu cell line to create an improved version that stably expresses *MyRF*, named Oli-neuM [12]. The Oli-neuM cell line, unlike Oli-neu, differentiates until the axonal engagement stage [12,96]. In the Oli-neuM cell line, 1280 molecules from the “Prestwick Chemical Library^®^ Food and Drug Administration (FDA) approved drugs” were repurposed at three doses, with OPC differentiation and MBP increase as the endpoints. This screen led to the identification of Clobetasol, Halcinonide, Medrysone, Ketoconazole and Clotrimazole as potent promyelinating drugs, in addition to Gefitinib and Erlotinib (EGF receptor inhibitors). Azathioprine, Itraconazole and Bromocriptine were among the most potent hits of this screen [12]. Since the top-ranking drugs were glucocorticoids regulating either Glucocorticoid Receptor (GR) and/or Smo activity (Clobetasol, Flurandrenolide, Medrysone, Halcinonide) and among the others were Ketoconazole and Itraconazole, that also bind Smo [21], it was concluded that the most potent promyelinating drugs identified in this screen target either GR or Smo, or both [12,97].

Deshmukh and colleagues (2013) used rat OPCs seeded in 96-well plates with micropillars, mimicking axons, to screen ~100,000 structurally diverse molecules. This phenotypical screen led to the identification of several compounds (Enprofylline, trans-RA, Fasudil), with the leading hit being Benztropine. Benztropine induces robust differentiation of rat and mouse OPCs and promotes remyelination in PLP1-induced Experimental Autoimmune Encephalomyelitis (EAE) [15].

Lee and colleagues (2012) observed that oligodendroglial cells preferentially myelinate axon fibres of large diameter (2–4 µm), also in the absence of neuronal input [98]. This led to the development of in vitro tests that allow the use of electrospun Polystyrene (PS) or poly(L-lactic acid) (**PLLA**) to mimic axons (synthetic axons) [98,99]. Micropillar 96-well platforms and chambers containing parallel electrospun PS filaments were widely used in primary or post-screening validation assays [8]. Based on these findings, Mei et al. (2014) developed micropillar-based platforms (fused-silica cones), which could be viewed in-plane for imaging the multi-layered membrane compaction around the cones, with the goal of identifying drugs that more specifically promote myelin sheath formation [16]. The development of cell culturing techniques suitable for the use of mouse epiblast-derived OPCs combined with the use of micropillar-based technology gave rise to a series of drug repurposing opportunities (Table 1) [16,17,100]. Although providing the potential for rapid comparative analysis of various conditions on myelin production, patterned glass cones do not have the cylindrical geometry of biological axons and cannot be used to measure the ability of OLs to laterally extend and to form Nodes of Ranvier [101].

These screens led to the identification of compounds characterized by the ability to block three enzymes in the cholesterol biosynthetic pathway (EBP, CYP51, S14R), whose inhibition leads to the accumulation of 8,9-unsaturated sterol [9,16,17,30,102]. In all cases, potential hits were further validated in toxically induced demyelination models (Lysolecithin or Cuprizone) and/or in the EAE animal model for inflammatory demyelination diseases [103].

Independently of the drug library or cell-based assay used, network and experimental analyses of selected promyelinating hits indicated redundancy in drug structural composition and targets [3,10,97]. Supporting this view, recent observations indicated that 5% of the identified compounds share the ability to induce 8,9-unsaturated sterol accumulation [3,10]. A second large set of promyelinating drugs comprises glucocorticoids sharing the ability to target the GR and Smo or drugs that inhibit Smo/Gli1 signalling, like GANT61, SANT-1, and GSA-10 (Table 1) [9,11,12,17,97,104]. A third group is more heterogeneous and includes RA agonists, Histamine H3 receptors, and Muscarinic receptor antagonists (Table 1) but also seems to have EBP or CYP51 as secondary targets. Several of these compounds have entered clinical trials, with Clemastine being the most promising (Table 2). However, none have been approved for clinical use in CNS remyelination therapy yet [2,4,105]. Given the redundancy of primary and secondary targets identified in phenotypical screens, the fact that the same compounds were repeatedly selected independently from the cellular models and drug library used and that their pairwise combination showed no additive effects [3,9] means it is possible that, under the conditions used so far, the drug targets of promyelinating drugs are saturated.

We are at a crossroads in choosing the next steps in developing more efficient drugs. An analysis of the data suggests that the high attrition rate of compounds in clinical trials (Table 2) could be attributed to multiple factors: (I) heterogeneity in the presentation of MS disease; (II) lack of understanding of the fundamental molecules and pathways leading to remyelination in healthy versus pathological conditions; (III) poor representation of the environment of MS disease in vitro cellular models in which phenotypical screenings were performed; (IV) lack of animal models representative of MS and other demyelination pathologies; (V) poor understanding of drug targets and secondary pathways activated during neural precursor differentiation and remyelination; and (VI) lack of markers to properly stratify patients in trials. A combination of these factors could also be an issue. Screening procedures should take into account the idea that the inflammatory environment surrounding the injury limits the differentiation of OPCs in patients with SPMS is widely accepted [4,83,86].

The combination of the use of AI and iPSCs obtained from patients samples represents a powerful and innovative approach to improving the discovery and development of drugs, especially in the context of myelin dysfunction. The following review highlights key technologies and approaches potentially useful to improve remyelination treatments in MS.

## 3. In Silico Models for Clinical Diagnosis and Drug Screenings

In recent years, computational science, and more specifically AI technology, have undergone significant advances and have seen general application, being used in every scientific field for various purposes, both in industry and academia [106]. The use of AI to understand the multiple aspects of MS pathology was advocated to solve several aspects that could help, from patient stratification to virtual drug screening.

### 3.1. Artificial Intelligence-Driven Methods to Improve Multiple Sclerosis Diagnosis and Patient Stratification

As it continues to innovate, the potential of AI in medicine and research expands, resulting in great improvements in diagnostic accuracy, disease monitoring, treatment optimisation, and drug discovery [107]. Integration of AI algorithms and tools into the clinical workflow is effective and useful in obtaining insights from patient data, improving the quality of treatment personalization, reducing costs, and optimising the health system performance [108]. In particular, the involvement of AI tools already had a significant role in drug research for diseases such as Alzheimer’s and some forms of cancer [106].

The main approach of AI in medicine is the participation of Machine Learning (ML), defined as a system capable of identifying patterns from input data to produce predictions from a new dataset, without the need to be taught or programmed manually. Through this tool, it is possible to extract information from patient data and identify correlations that could not be seen with the naked eye [108], to assess the disease stage, and to better personalize the treatment. ML is also involved throughout the entire drug discovery process, to find a new use for already existing drugs, predict drug–protein interactions, or study the bioactivity of molecules [106]. AI algorithms can be useful in diagnosis optimization, treatment personalization, and drug discovery for MS [109].

Generally, MS diagnosis is assessed by a combination of Magnetic Resonance Imaging (MRI) data, Cerebrospinal Fluid (CSF) analysis, neurological examination, and motor-sensory-evoked potentials [110]. However, the diagnosis of MS is difficult as its characteristic symptoms are common to many other diseases, leading to several misdiagnoses [111]. For this reason, the analysis of clinical data through the aid of AI tools and algorithms might be of enormous value. Nabizadeh et al. (2022) systematically reviewed a total of 38 studies that used AI algorithms during MS clinical diagnosis, 34 of them with an accuracy greater than 80%. These studies foresaw the application of ML algorithms or other AI-based strategies in the analysis of clinical data such as MRI, Optical Coherence Tomography (OCT), CSF biomarkers, movement analysis, and other factors, resulting in improved diagnostic specificity, sensitivity, and accuracy [112].

The aid of AI is not limited to MS diagnosis but can also be used to assess the stage of the disease. Based on MRI images of patients, it is possible to use Convolutional Neural Networks (CNNs), an ML model, to analyse the volume of brain lesions and assess the disease stage [113]. Similarly, Dwyer and colleagues (2021) developed the so-called Deep-Gray Rating, a deep learning algorithm that studies T2-FLAIR MRI images to calculate thalamic atrophy, generally correlated with cognitive decline and disease progression [114]. Acquaviva et al. (2020) designed an ML workflow that helps to discriminate between healthy subjects and patients and to determine the different stages of the disease (CIS, RRMS, PPMS, and SPMS) based only on the gene profile of Peripheral Blood Mononuclear Cells (PBMCs) of the subjects [115]. Differently, the ProMiSi project applied several ML algorithms and models on data from patients (demographic information, treatments, specific tests) to assess MS progression and severity, obtaining more than 80% accuracy [116]. Even the analysis of patients’ movement behaviours through AI approaches can result in improvements in the diagnosis and the assessment of the disease stage. These data, which provide information about the patient’s gait disturbances, are recorded using specific treadmills [117], or making patients pass on instrumented walkways [118]. Other elements that can be analysed through ML models to measure disease progression are cognitive impairment tests [119,120], and voice recordings [121]. Through these numerous methods, it is possible to improve the efficacy in the diagnosis and in the MS disease stage assessment, which is essential to ameliorate treatment personalization and optimization.

In addition to diagnosis, AI is fundamental also for the selection and development of new treatments. An example is the expanding field of nanorobotics, where AI tools are widely used, possibly to find effective ways to cross the BBB and target the lesions [122]. In the field of treatment selection and identification, it is worth mentioning the use of Digital Twins (DTs) technology [123]. DTs employs an AI-based analysis of several disease parameters—including clinical and paraclinical outcomes, multi-omics, biomarkers, patient-related data, information about the patient’s life circumstances and plans, and medical procedures—to create a virtual twin of the patient that can be used to simulate personalized treatment and visualize disease progression [123].

The use of AI models and techniques has the potential to improve the diagnosis, stratification, and treatment of MS patients, in addition to reducing timing and costs.

### 3.2. Drug Repurposing through In Silico Binding

A modern and promising approach towards progress in drug discovery for remyelination is in silico drug repurposing, a strategy that consists of the identification of novel targets for existing drugs through in silico simulations. Finding drugs already known to tackle a different disease could be essential due to reduced costs and risks [124]. An example of repurposed drugs for MS treatment is dimethyl-fumarate, previously prescribed for psoriasis and recently adapted for RRMS treatment [125], while other antipsychotic drugs, such as Clemastine, Benztropine, and Quetiapine were identified as remyelinating [16], and are currently in different Clinical Trials for MS treatment (Table 2).

Mihai et al. (2019) performed an in silico screen of 903 repurposable drugs selected from DrugBank to target the Transient Receptor Potential Ankyrin 1 (TRPA1), which is involved in calcium levels regulation, synapse inhibition efficacy, and Long-Term Potentiation (LTP) [126,127], and known to be a possible target for MS treatment [128]. In the study, the group developed a predictive algorithm model to discover TRPA1 antagonists, using the most common in silico techniques (data mining, classification and regression Quantitative Structure–Activity Relationships (QSAR) modelling and molecular docking) [126]. The identified TRPA1 inhibitors were subsequently analysed and ranked for their ability to pass the BBB, resulting in only 10 molecules able to cross the BBB and with a TRPA1 binding probability greater than 90%. Of these, they selected Desvenlafaxine (O-desmethyl venlafaxine), Paliperidone (9-hydroxy risperidone), and Febuxostat for potential repurposing for MS treatment [126].

Sardari et al. (2023) screened 263 drugs using in silico binding, targeting the Sphingosine 1-Phosphate receptor Lyase (S1PL) receptor and Cyclophilin D (CypD) [129]. S1PL is a lyase that binds to the S1P receptor to catalyse sphingolipid metabolism, while CypD is a mitochondrial enzyme that catalyses proline isomerisation in proteins. Both were previously indicated as possible targets for MS treatment [130,131]. The group used the Lamarckian Genetic Algorithm (LGA) of AutoDock 4.2 software to run docking simulations between the 263 drug candidates and the S1PL and CypD enzymes [129]. The binding performances were evaluated according to the free binding energy (ΔGb) between the interacted targets: the drugs with better docking scores for both targets were the antipsychotics Zuclopenthixol and Lurasidone [129], suggesting their potential for repurposing for MS.

The stability of the molecular interaction of these two drugs and their targets was analysed using a computational approach, using GROMACS 5.1.1 software to perform all-atom Molecular Dynamics (MD) simulations. Through Root-Mean-Square Deviation (RMSD), Root-Mean-Square Fluctuation (RMSF), Rg, intermolecular, and intramolecular H-bond analysis, the Lurasidone-CypD complex was found to be the most stable [129].

Together, these studies provide examples of the great potential of in silico analysis of drug-target binding for repurposing already existing drugs for MS treatment. The identification of other targets and using different classes of repurposable drugs could be an invaluable asset for MS drug discovery.

## 4. Disease-Relevant 2D and 3D Models for Drug Screenings and Physio-Pathological Studies

It is widely acknowledged that the clinical translation of preclinical molecules is difficult due to the lack of reliable disease-relevant models for demyelination and remyelination. This is particularly true for MS pathology as oligodendrocyte precursors for drug screening and validation studies rely mainly on animal-derived samples. To create models for large drug screening, it is essential to identify the main actors that regulate remyelination in healthy conditions in the adult brain [4].

Access to primary human CNS tissue samples is limited to post-mortem and biopsied tissue, making iPSCs-derived neural cells a potential alternative source to CNS neural cell types that possess the full genetic background of the disease of interest, allowing for the establishment of a clear sequence of pathological events [132]. Thus patient-derived iPSCs have emerged as a potential tool to overcome the scarcity of human CNS samples and the low representation of rodents for pathophysiological studies.

iPSCs-derived cells, initially pioneered by Shinya Yamanaka and his team [133], were widely used to clarify some aspects of CNS genetic pathologies and pharmacological studies due to their ability to differentiate into various brain cell types, mimicking the developmental trajectory seen in vivo [132,134].

iPSCs-derived neural cells are ideal for studying the genetics of demyelinating disease; however, they are less suitable for those diseases in which the imprint of lifestyle and environmental factors, in the form of epigenetic modifications, drive the pathology, as these features are lost during reprogramming. Moreover, iPSC technology requires a comprehensive transcriptomic and genotypic variability analysis, which poses a significant bottleneck in its application for drug discovery in academic settings where these technologies are less easily available [132].

Various MS patients’ cell types were used to generate iPSCs: PBMCs, fibroblasts, mesenchymal cells, and renal proximal tubule epithelial cells [135]. In the case of MS patients’ samples, one to eight cell lines were considered for each study. The different experimental settings and starting cell types have so far failed, at least for this pathology, to draw a clear conclusion on which is the minimal variability to be considered in MS studies using iPSCs. Even if various methods were developed for the use of primary cells from MS patients, the lack of well-characterized patient samples (i.e. samples that were thoroughly studied and described) and the lack of standardised protocols for converting iPSCs into OPCs have limited the possibility of comparing results among studies [132]. The rapid establishment of biobanking of patient-derived iPSC samples is essential to accelerate drug discovery programs at the academic level [86,132,136]. Despite these limitations, iPSC-derived NPCs and OPCs remain a good, promising model for studying demyelinating pathologies.

### 4.1. Generation of Oligodendrocyte Precursor Cells from Induced Pluripotent Stem Cells

Currently, several protocols are available to obtain oligodendrocytes in two-dimensional (2D) and three-dimensional (3D) organoid systems [137]. Despite extensive studies modelling oligodendrocytes, development in 2D and brain organoids is still challenging.

**Figure 1:**
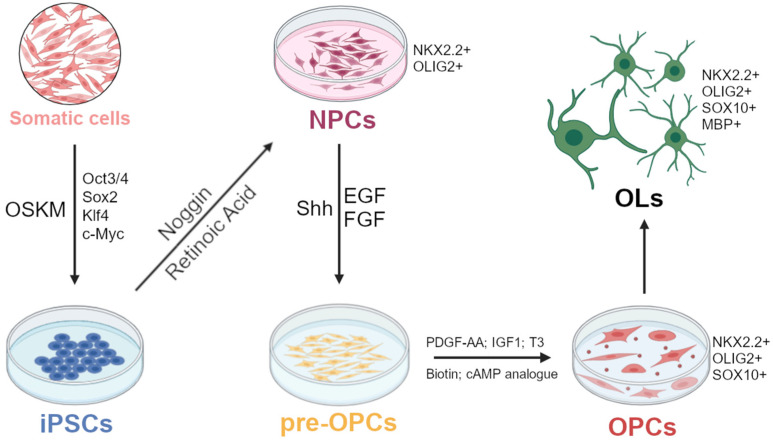
**Typical protocol for the generation of OPCs from patients’ iPSCs.** Somatic cells obtained from patients are reprogrammed into induced Pluripotent Stem Cells (iPSCs) using the Yamanaka transcription factors (*Oct3/4, Sox2, Klf4, c-Myc*). iPSCs can be then directed towards the neural lineage through the application of Noggin and Retinoic Acid (RA). These cues guide iPSCs to become Neural Precursor Cells (NPCs), further fated to the Oligodendrocyte Precursor Cells (OPCs) linage under the influence of factors such as Sonic Hedgehog (Shh), Epidermal Growth Factor (EGF) and Fibroblast Growth Factor (FGF). Pre-OPCs can reach the OPCs stage through a glial factor-medium enriched with PDGF-AA, IGF1, T3, biotin and a cAMP analogue.

The transition from iPSCs to OPCs occurs in several meticulously scheduled stages. Initially, somatic cells like fibroblasts or PBMCs are reprogrammed using plasmids carrying transcription factors such as Cellular myelocytomatosis oncogene (*c-Myc*), Octamer-binding transcription factor 3/4 (*Oct3/4*), SRY-box 2 (*Sox2*), and Krüppel-like factor 4 (*Klf4*) (Figure 1) [138].

Key steps of the generation of Neuro-Epithelial Cells (NECs)/NPCs from iPSCs are the co-expression of *OLIG2* and NK2 Homeobox 2 (*NKX2.2*) and the subsequent expression of the transcription factor *SOX10*, which marks the oligodendrocyte lineage [139]. *NKX2.2* can be induced by treating Embryonic Stem Cells (ESCs) with the caudalization factor RA [140]. The Noggin-mediated inhibition of the BMP signalling pathway is necessary for the development of mature ramified oligodendrocytes expressing *MBP* [141], since inhibitory effects on the oligodendrocyte populations can be mediated by activation of proteins in the BMP pathway [142]. These principles paved the basis for further development of protocols allowing to obtain mature OLs from iPSCs-derived OPCs.

Human ESCs can also be induced to differentiate into Paired Box 6 (PAX6) and SRY-box 1 (SOX1) positive NECs or NPCs through the generation of Embryoid Bodies (EBs), as aggregates in suspension. Treatment with morphogen Shh and RA can produce ventral and caudal patterning, respectively. The removal of RA prevents the differentiation of these cells into motor neurons, physiologically originating from the same embryonic domains [137,143].

The pre-OPC pool increases with FGF2 application because of its mitogenic effect on NPCs. Pre-OPCs are selected as successful if they co-express the transcription factors *OLIG2* and *NKX2.2*. However, to promote the pre-OPCs transition to OPCs, FGF2 must be removed since FGF2 represses OPC specification [143]. The pre-OPC differentiation into OPCs can be achieved through a glial factor-containing medium (PDGF-AA, Insulin-like Growth Factor 1 (IGF1), a cyclic AMP (cAMP) analogue, biotin and triiodo-L-thyronine (T3)) that promotes the survival and proliferation of OPCs. This protocol allows for the production of OPCs in 90 days. However, the use of RA as part of the differentiation protocol most likely leads to OPCs with characteristics similar to those of the spinal cord, which are functionally and transcriptionally different [144,145].

In the past, Goldman’s group established a 150-day culture reprogramming protocol to successfully obtain iPSC-derived oligodendrocytes based on samples from three different individuals [139]. These steps include the generation of EBs and a later application of Purmorphamine (PM) and RA for ventral and caudal patterning, respectively. This induces the appearance of OLIG2+ and NKX2.2+ cells, which are known as early pre-OPCs [146]. The transition of pre-OPCs to late pre-OPCs was promoted by treatment with FGF2 and PM and removal of RA. The glycogenic spheres thus formed were dissociated and plated in a glial medium for 3–4 months resulting in an OPC population that was sufficient for both in vivo and in vitro studies. They also obtained oligodendrocytes positive for O4 and MBP in vitro, capable of myelination when co-cultured with human foetal cortical neurons. Later, they transplanted OPCs into shiverer mice with a 4-month culture period, resulting in the production of compact myelin and robust myelination in the mouse forebrain, meanwhile showing a prolonged life expectancy [139].

Another method to produce oligodendrocytes from iPSCs in adherent cultures is through the indirect inhibition of Suppressor of Mothers Against Decapentaplegic (SMAD) via treatment with LDN-193189, which acts on the BMP pathway, and SB431542, which acts on the Transforming Growth Factor-β (TGF-β) pathway [147,148,149]. With this method, OLIG2 precursor cells were produced using RA treatment at the start of differentiation. In later studies, OLIG2+ cells were produced using dual-SMAD inhibition with RA while the Shh pathway was stimulated by Smoothened agonist (SAG), without the use of PM. This approach allowed early detection (50 days) for O4+ cells, with an increased population up to day 75, halving the time required to complete the protocol [139].

### 4.2. Use of Induced Pluripotent Stem Cell-Derived Oligodendrocyte Precursor Cells to Study Demyelinating Diseases

The applications of iPSC-derived cells range from fundamental research to therapeutic strategies. iPSCs-derived cells were used to examine the genetic factors underlying the development and progression of demyelinating diseases by generating reporter lines and disease-specific mutations, thanks to advances in genome-wide analyses and genome editing tools like Clustered regularly interspaced palindromic repeats (CRISPR)/Cas9 [136,150,151,152]. The iPSC technology was also used to generate genetic variants of interest that led to a more fruitful understanding of the pathological features of the disease [152]. Transplanting iPSC-derived OPCs has shown potential in preclinical models of demyelinating disorders by increasing remyelination and functional recovery [153].

Early research on MS-iPSC-derived NPCs indicated that cells experience premature cellular senescence and are less capable of providing neuroprotection than NPCs derived from healthy controls. In these protocols, and after two weeks of Cuprizone feeding, mice received injections of PPMS or control iPSC-derived NPCs into their veins. These animals were fed with Cuprizone for an additional two weeks before tissue collection when CC of MS-NPC-injected mice had poorer myelination and a higher number of apoptotic cells than the control [7]. Interestingly, NPCs in this study were more likely to develop into astrocytes than oligodendrocytes, showing a low ability to promote remyelination. This shows that NPCs derived from PPMS iPSCs have an inherent inability to develop into OLs and do not help myelin repair through other means. Paracrine signalling of NPCs derived from PPMS iPSCs is also disrupted, as cultivating primary rat OPCs from the cerebral cortices of neonatal rat pups with conditioned media produced from PPMS iPSCs improves their vulnerability to glutamate-induced cell death and inhibits the development of oligodendrocyte development [136].

Furthermore, Kawabata et al. (2016) used iPSC-derived OPCs to study genetic defects leading to demyelination. They managed to obtain more facilitated differentiation of the iPSCs-induced oligodendrocyte lineage, controlled by immunohistochemical studies for differentiation markers [146]. During the early phase of EB formation, those treated with the DSB mix (Dorsomorphin (a BMP signal inhibitor), SB431542, and BIO (a GSK3 inhibitor)) were driven to enhance differentiation into NSCs. Reverse Transcription-quantitative Polymerase Chain Reaction (RT-qPCR) analysis of the expression of the NSCs marker *SOX1* in EBs revealed a significantly higher NSCs induction efficiency in their protocol with the DSB mix compared to previously established methods (control, DSB) and dual-SMAD inhibition. They added RA for caudalization and PM for ventralization during EB development and dissociation. These modifications and approaches generated Pelizaeus-Merzbacher Disease (PMD)-specific human iPSCs from two individuals with varying clinical severity and *PLP1* missense mutations. Two mutations, one in the transmembrane domain (PMD1) and one in the extracellular domain (PMD2), differ from those seen in earlier PMD animal models. In addition, they created customized OLs for each patient. This model examines the relationship between PMD’s molecular pathophysiology and cell biology, including differentiation, myelination, and apoptosis, in patient-derived live OLs using morphological, biochemical, and molecular approaches. Conventional disease models were inadequate for these evaluations [146,154].

iPSCs-derived neural cells are very useful for understanding the effects of promyelinating drugs in an MS-genetic background. Promyelinating drugs such as Benztropine, Miconazole, and Clemastine failed to restore differentiation of human iPSCs-derived oligodendrocytes (hiOLs) in the presence of PBMCs supernatants. Instead, immunomodulatory treatment of PBMCs partially restores hiOLs differentiation, showing that the blockage of oligodendrocytes differentiation in RRMS is caused by an extrinsic inflammatory environment rather than intrinsic oligodendroglia factors. This study, in addition of showing that promyelinating drugs selected in a non-inflammatory environment might act differently in an inflammatory environment, such as MS, demonstrates that iPSC-derived OPCs can be used to clarify the basis of a demyelinating event in a pathological environment [86]. Unfortunately, we still lack standardised procedures and protocols for generating OPCs from MS patient-derived iPSCs.

### 4.3. Organoid-Derived Models for the Screening of Remyelinating Drugs

Human stem cell-derived brain organoids are proving to be an ideal model system for studying neurological diseases, particularly those that involve myelination defects [155]. Three-dimensional cerebral organoids (also called spheroids) cultures derived from iPSCs were developed and are suitable cell-based models for the investigation of developmental and neurodegenerative diseases [156,157]. Since organoid systems of the human brain mimic the in vivo 3D make-up of the brain, they represent an opportunity to investigate brain functions in health and disease conditions. However, current protocols for generating brain organoids with sufficiently mature oligodendrocytes that deposit myelin on endogenously produced neurons are time-consuming and complicated [155]. Only after a prolonged glial differentiation protocol of 210 days, a relatively robust induction of oligodendrogenesis and myelination in organoids is reached, but Kim et al. (2019) established a protocol that accelerates the specification of OLs into organoids and shortened the protocol to 105 days of culture [155,158]. Therefore, a human pluripotent stem cell line expressing the early oligodendroglial gene *SOX10* was created; the cell line was used to develop a facile and rapid 42-day protocol for the generation of human brain organoids that contain oligodendrocytes capable of myelinating endogenously co-specified cortical neurons and supporting astrocyte differentiation. This protocol results in a fast method to generate mature oligodendrocytes within a human cortical organoid [155].

Considering the scarcity of advances in neuroectoderm-derived human CNS white matter research, a protocol was developed to generate neuroectoderm-derived embedding-free human brain organoids enriched with oligodendrocytes, bypassing one of the limitations of the organoids themselves. In this model, the development of a necrotic core is rarely observed, which generally is formed during months of in vitro culture of brain organoids. In addition, single-cell RNA sequencing of the oligodendrocyte brain organoid was performed to compare the oligodendrocytes generated with those developed in vivo in the human foetal brain. In addition to sequencing, electrophysiological studies should be necessary because they are fundamental for understanding the functional maturity of these cells [159].

One of the main limitations of the brain organoids is that microglial cells are not co-produced within the organoid, because of their non-neural lineage derivation. However, they could be generated separately and seeded via co-culture with brain organoids [160].

However, Kim and Jiang (2021) provided a protocol for generating fused forebrain organoids derived from human pluripotent stem cells that rely on the fusion of dorsal and ventral forebrain organoids, resulting in myelination, so MBP+ cells. These fused organoids develop mature oligodendrocytes in three weeks. The diameter of these organoids is typically approximately 1.2 to 2.0 mm after fusion, and they are visible to the naked eye. The specification of brain regions can be easily monitored using OLIG2-Green Fluorescent Protein (GFP) cell lines during the patterning process under the epifluorescence microscope, as well as by RT-qPCR with region-specific markers for the ventral forebrain, such as *NKX2.2*, Distal-Less Homeobox 1 (*DLX1*), LIM Homeobox 6 (*LHX6*), and for the dorsal forebrain, such as Empty Spiracles Homeobox 1 (*EMX1*) and T-box brain Transcription Factor 2 (*TBR2*) [160].

Furthermore, Yun et al. (2020) showed that 3D cultures of *OLIG2* and *NKX2.2* expressing neurospheres efficiently produced mature astrocytes and oligodendrocytes in terms of morphologies and expression patterns, recapitulating the native 3D environment. Thus, robust populations of OLs were generated at week 8 of the culture of spheroids treated with promyelination drugs, such as Benztropine and Miconazole. Benztropine-treated spheroids showed similar results to T3-treated spheroids, while Miconazole-treated spheroids exhibited elevated MBP+/SOX10+ populations and abundant MBP+ spheroids. In addition, Magnetic Activated Cell Sorting (MACS)-purified O4+ oligodendrocytes in Miconazole-treated spheroids were engrafted into *MBP*-deficient shiverer mice. These transplanted cells resulted in MBP+ oligodendrocytes and contributed to myelin compaction after 12 weeks of transplantation [157].

As previously mentioned, iPSCs lines from MS patients can be created with patient fibroblasts reprogrammed by retroviral delivery or by the mRNA/miRNA method but also from blood cells using Sendai virus or from renal epithelial cells transfected with episomal factors. Cerebral organoids were developed from iPSCs derived from MS patients’ blood samples. They guarantee the consequences of the effect of the patient’s genetic background on neural cells and their interactions in a model free of immune system interaction and within a controlled microenvironment. Since cerebral organoids do not contain blood vessels or immune cells, they do not reproduce the inflammatory demyelination classically associated with MS [156]. New protocols were established to generate vascularised organoids, such as the one developed by Wimmer et al. (2019) [156,161].

The absence of immune cells in brain organoids would limit the use of the model to study immune–viral interactions. Nevertheless, brain organoids were used as a model to study the effects of the John Cunningham Virus (JCV), a circular DNA polyomavirus that produces Progressive Multifocal Leukoencephalitis (PML). This virus infects human glial cells and can cause massive destruction of oligodendrocytes and astrocytic abnormalities as well as demyelination, leading to the development of PML and eventually death. Therefore, the 3D human brain organoid was used as a model to study JCV infection of the human brain. It should prove useful for screening therapeutic compounds as well as examining diverse pathogenic mechanisms [162].

Another model of iPSC-derived myelinating organoids (‘myelinoids’) was created with a ventral–caudal cell fate from EBs generated from dual-SMAD inhibition and RA and SAG treatment to promote caudalization and ventralization, respectively. Myelinoids were used to study oligodendrogenesis through compact myelin formation and axon organization and for pharmacological assessment of myelination. Myelinoids showed compact myelin formation and organisation of the myelinated axons; therefore, representing a useful tool for studying a monogenetic disease of myelinated axons (Neurofascin-155 deficiency) using patient-derived cells, caused by the impaired formation of the paranodal axoglial junction formation [163].

iPSCs derived from MS patient’s samples can be differentiated into mature astrocytes, oligodendrocytes, and neurons with normal karyotypes [156]. Daviaud et al. (2023) managed to derive cerebral organoids from iPSCs of healthy control subjects as well as from patients with PPMS, SPMS, and RRMS to achieve a clearer picture of the pathological basis of the varied clinical phenotypic expressions of MS [156]. In MS organoids, particularly in PPMS, a decrease in the proliferation marker Ki67 and a reduction in the SOX2+ stem cell pool are associated with an increased expression of neuronal markers COUP-TF-interacting protein 2 (*CTIP2*) and T-box brain Transcription Factor 1 (*TBR1*), as well as a strong decrease in oligodendrocyte differentiation. These findings show that the genetic background of a patient can directly alter stem cell function, providing new information on innate cellular dysregulation in MS. Furthermore, these results highlight a defect of oligodendrocyte differentiation and maturation in MS organoids, suggesting that the remyelination capacity might be diminished in patients with PPMS. In any case, cerebral organoids mimic the development of the human brain, while MS occurs around 20–40 years old. 

In conclusion, iPSC-derived organoids represent a very helpful tool for better understanding the pathogenesis of MS before the onset of the disease. Some recently improved protocols lead to very substantial organoid development, with a variability that is close to the human brain one [156]. The existence of efficient and rapid protocols to create brain organoids makes them a promising and flexible platform for modelling CNS diseases associated with hypomyelination or demyelination as well as for functional neuro-genomics studies and drug screening [155], thus guiding toward personalized medicine [157].

## 5. Conclusions

Remyelination therapies offer hope to millions of patients affected by demyelinating diseases to regain lost functions. However, we now find ourselves at a crossroad, uncertain of the best path to reduce the high attrition rate of these drugs. This challenge is at the forefront of current discussions. New technologies offer exciting opportunities to create new screening methods and improve existing ones. With the power of AI, we can deepen our research into drug libraries and their results, paving the way for more effective treatments. The integration of AI, iPSCs and organoid research holds great promise for discovering more effective drugs. However, current iPSC technologies face challenges such as phenotypical variability, limited performance and long protocols for producing myelinating oligodendrocytes. Furthermore, high-throughput organoid imaging and data extraction require advanced microscope techniques. AI algorithms are poised to improve the number of data that can be extracted from image analyses and combined with clinical evaluation of patient medical data. This will allow for better patient stratification, which will lead to improved diagnosis and treatment through comprehensive multi-source clinical data and follow-up. The creation of biobanks containing iPSC samples obtained from demyelination pathologies, as well as the standardization of protocols for generating precursor cells of neurons and oligodendrocytes will increase the global access of these technologies to researchers. Organoids derived from iPSCs will provide a deeper understanding of the complex interactions between glial cells and neurons during the progression of demyelinating diseases and will significantly advance our understanding of the genetic basis of diseases affecting myelin.

## Figures and Tables

**Table 1:** List of promyelinating compounds targeting enzymes of the sterol pathway and/or Smoothened (Smo) receptor. Targets of interest: drug target involved in remyelination, as Emopamil Binding Protein (EBP), Smo, Sterol 14-demethylase (CYP51), and Sterol C-14 reductase (S14R).

Compound	Primary Targets	Targets of Interest
Bazedoxifene	Estrogen receptor [3,14]	EBP [3]
Benztropine	Muscarinic receptor [15]	EBP [9]
Clemastine	Histamine/muscarinic [3,16]	EBP [9]
Clobetasol	Glucocorticoid Receptor (GR) [17,18]	Smo [12,19]
Clotrimazole	Fungal CYP51 [20]	CYP51 [9,20], Smo [21]
Donepezil	Acetylcholinesterase [3,22]	EBP [3]
Fluphenazine	Antipsychotic; multiple receptors [3]	EBP [3]
GSA-10	Smo [11,23]	Smo [11,23]
GSK239512	Histamine H3 receptors [24]	EBP [3]
Halcinonide	GR [19]	Smo [12]
Hydroxyzine	Histamine receptors [3]	EBP [3]
Ifenprodil	N-methyl-D-aspartate (NMDA) receptors [3,25]	S14R [3]
Itraconazole	Fungal CYP51 [20]	CYP51 [20], Smo [21,26,27]
Ketoconazole	Fungal CYP51 [3,21]	CYP51 [3], Smo [9,21,26,27]
Medroxyprogesterone acetate	Progesterone receptor [3,14]	CYP51 [9]
Megestrol	Progesterone receptor [3,14]	CYP51 [3]
Miconazole	Fungal CYP51 [17]	CYP51 [9], Smo [21]
Quetiapine	Atypical antipsychotic [3]	EBP [3]
Raloxifene	Estrogen receptor [3,14]	EBP [3]
Ro 25-6981	NMDA receptors [3]	S14R [3]
Salmeterol	Adrenergic receptors [3]	S14R [3]
SANT-1	Smo [10,27,28,29]	Smo [10,27,28,29]
Tamoxifen	Estrogen receptor [3,14]	EBP [9]
Toremifene	Estrogen receptor [3,14]	EBP [3]
U50488	k-opioid receptor [3]	EBP [3]
Vanoxerine	Multiple ion channel families [3]	EBP [3]
Vesamicol	Vesicular acetylcholine transport [3,30]	EBP [3]

**Table 2:** Compounds in clinical trials as of 19/06/2024 (https://www.clinicaltrials.gov/, accessed on 19 June 2024). Trials were filtered with the following keywords: “Multiple Sclerosis” (Condition/disease), “Remyelination” (Other terms).

Compounds	Targets	ClinicalTrials.Gov ID	Trial Stage	Details [31]	Trial Status
Liothyronine	Thyroid hormone receptor alpha and beta [32]	NCT0276005	1	**Thyroid Hormone for Remyelination in Multiple Sclerosis (MS): A Safety and Dose Finding Study (MST3K).** This is a phase 1 study evaluating the safety and maximum tolerated dose of Liothyronine (T3) in subjects with multiple sclerosis.	Completed (October 2017)
BIIB061	Tau [33]	NCT02521545	1	**Single-Dose Study of a New Formulation of BIIB061.** The primary objective of the study is to assess the pharmacokinetic (PK) profile of BIIB061 in the new oral formulation in the fasted state in healthy male and female volunteers.	Completed (August 2015)
BIIB033 (Opicinumab)	Leucine-rich repeat and Immunoglobin-like domain-containing protein 1 (LINGO-1) [34]	NCT01244139	1	**Safety Study of BIIB033 in Subjects with Multiple Sclerosis.** The main purpose of the study is to evaluate the safety, tolerability, and pharmacokinetic profile of two intravenous infusions of BIIB033 administered two weeks apart in subjects with MS.	Completed (April 2012)
Natalizumab	Integrin alpha-4 [32]	NCT00559702	1	**Safety Study of Natalizumab to Treat Multiple Sclerosis (MS).** The primary objective of this study is to compare the pharmacokinetic and pharmacodynamics of single subcutaneous and intramuscular doses of 300 mg natalizumab to intravenous administration of 300 mg natalizumab in MS participants.	Completed (November 2011)
Olesoxime	Mitochondrial permeability transition pore [32]	NCT01808885	1	**Safety Study of Olesoxime in Patients With Stable Relapsing–Remitting Multiple Sclerosis Treated With Interferon Beta. (MSREPAIR).** This is a 24-week phase 1b, randomized, double-blind, placebo-controlled, parallel-group, multicentre safety study comparing the tolerance profile of olesoxime (495 mg, od) when administered on top of Interferon beta in patients with stable RRMS.	Completed (January 2014)
Metformin	Electron transfer flavoprotein-ubiquinone oxidoreductase, 5′-AMP-activated protein kinase subunit beta-1 [32]	NCT05893225	2	**Metformin Add-on Clinical Study in Multiple Sclerosis to Evaluate Brain Remyelination and Neurodegeneration.** This clinical trial aims to demonstrate that metformin can prevent clinical disability in patients with progressive MS by stopping or slowing down neurodegeneration by enhancing endogenous remyelination.	Recruiting
Ifenprodil	Glutamate receptor ionotropic, N-methyl-D-aspartate (NMDA) 1 and 2B [32]	NCT06330077	2	**Ifenprodil as a ReMyelinating repurpOsed Drug in Multiple Sclerosis (MODIF-MS).** The preclinical research has allowed for the identification of ifenprodil as a powerful drug to promote myelin repair in vitro and in vivo across species.	Not yet recruiting
Metformin	Electron transfer flavoprotein-ubiquinone oxidoreductase, 5′-AMP-activated protein kinase subunit beta-1 [32]	NCT04121468	2	**A Phase I Double-Blind Study of Metformin Acting on Endogenous Neural Progenitor Cells in Children With Multiple Sclerosis.** A randomized multiple baseline feasibility trial where participants will start taking metformin at one of 3 randomly determined points (3 months, 6 months or 9 months) during the 12-month trial.	Unknown
Quetiapine	5-hydroxytryptamine receptor 2A, D2 dopamine receptor [32]	NCT02087631	2	**Safety and Tolerability of Quetiapine in Multiple Sclerosis.** The purpose of this clinical trial is to determine if extended-release quetiapine in a dose of 300 mg daily is tolerable to people with RRMS and progressive MS.	Completed (July 2019)
Metformin; Clemastine	Electron transfer flavoprotein-ubiquinone oxidoreductase, 5′-AMP-activated protein kinase subunit beta-1; Histamine H1 receptor [32]	NCT05131828	2	**CCMR Two: A Phase IIa, Randomised, Double-blind, Placebo-controlled Trial of the Ability of the Combination of Metformin and Clemastine to Promote Remyelination in People With Relapsing–remitting Multiple Sclerosis Already on Disease-modifying Therapy.** The goal is to establish whether the combination of metformin and clemastine can promote remyelination in people with MS.	Recruiting
Adrenocorticotropin	Adrenocorticotropic hormone receptor [32]	NCT00986960	2	**Effect of Adrenocorticotropin Injection With Weekly Interferon Beta in Patients With Relapsing–Remitting Multiple Sclerosis (MS) (ACTH).** The purpose of this study is to evaluate whether the use of ACTH in addition to Avonex is effective in the treatment of RRMS.	Withdrawn (December 2010)
BIIB033 (Opicinumab)	LINGO-1 [34]	NCT03222973	2	**Efficacy and Safety of BIIB033 (Opicinumab) as an Add-on Therapy to Disease-Modifying Therapies (DMTs) in Relapsing Multiple Sclerosis (MS) (AFFINITY).** The primary objective of Part 1 of this study is to evaluate the effects of BIIB033 versus placebo on disability improvement over 72 weeks.	Terminated (February 2021)
Metformin	Electron transfer flavoprotein-ubiquinone oxidoreductase, 5′-AMP-activated protein kinase subunit beta-1 [32]	NCT05298670	2	**Drug Repurposing Using Metformin for Improving the Therapeutic Outcome in Multiple Sclerosis Patients.** This study aims to evaluate the effect of Metformin as an add-on therapy for improving the outcome in RRMS patients.	Recruiting
BIIB061; Interferon-beta1; Glatiramer acetate	Tau [33]; Interferon alpha/beta receptor 1 [32]; Major Histocompatibility Complex [35]	NCT04079088	2	**Study to Evaluate Oral BIIB061 Added to Interferon-beta1 (IFN-β1) or Glatiramer Acetate in Relapsing Multiple Sclerosis (RMS).** The primary objectives of the study are to evaluate the safety of BIIB061 versus placebo in participants with RMS and to evaluate the efficacy of BIIB061 to improve disability outcome versus placebo in participants with RMS.	Withdrawn
Bazedoxifene acetate	Estrogen receptor [32]	NCT04002934	2	**Bazedoxifene Acetate as a Remyelinating Agent in Multiple Sclerosis (ReWRAP).** The primary goal of this study is to assess the efficacy of bazedoxifene (BZA) as a remyelinating agent in patients with RRMS.	Recruiting
CNM-Au8	Nicotinamide adenine dinucleotide reduced form (NADH) [36]	NCT03536559	2	**Nanocrystalline Gold to Treat Remyelination Failure in Chronic Optic Neuropathy In Multiple Sclerosis (VISIONARY-MS).** The objective of this trial is to assess the efficacy and safety of CNM-Au8 as a remyelinating treatment for vision-impairing MS lesions in participants who have chronic vision impairment as a result of RRMS.	Terminated (July 2022)
Domperidone	D2 and D3 dopamine receptors [32]	NCT02493049	2	**Pilot Trial of Domperidone in Relapsing–Remitting Multiple Sclerosis (RRMS).** The first major objective of this pilot trial is to demonstrate that it is possible to study myelin repair in relapsing–remitting multiple sclerosis (RRMS) patients with enhancing lesions on MRI by using advanced imaging techniques.	Completed (February 2019)
Testosterone	Androgen receptor [32]	NCT03910738	2	**TOTEM RRMS: TestOsterone TreatmEnt on Neuroprotection and Myelin Repair in Relapsing–Remitting Multiple Sclerosis (TOTEM-RRMS).** The trial sought to demonstrate the effect of testosterone supplementation in testosterone-deficient patients in a multicentre, randomized, parallel-group, double-blind, placebo-controlled trial. The main objective will be to determine the neuroprotective and remyelinating effects of testosterone using tensor diffusion imaging and thalamic atrophy analyses.	Recruiting
Clemastine	Histamine H1 receptor [32]	NCT02040298	2	**Assessment of Clemastine Fumarate as a Remyelinating Agent in Multiple Sclerosis (ReBUILD).** The main purpose of this study is to assess clemastine as a remyelinating agent in patients with relapsing forms of multiple sclerosis.	Completed (April 2016)
Phenytoin	Sodium channel protein type 1, 2, 5 and 8 subunit alpha, Potassium voltage-gated channel subfamily H member 2, Voltage-dependent L-type calcium channel [32]	NCT01451593	2	**Neuroprotection With Phenytoin in Optic Neuritis.** The purpose of this study is to determine whether phenytoin (which blocks sodium entry into cells) can protect against loss of nerve fibres and prevent loss of vision after optic neuritis.	Completed (March 2015)
GSK239512	Histamine H3 receptor [35]	NCT01772199	2	**Study to Assess Whether GSK239512 Can Remyelinate Lesions in Subjects with Relapsing–Remitting Multiple Sclerosis.** This is a randomized, parallel-group, placebo-controlled study designed to assess whether GSK239512 can enhance lesion remyelination in subjects with RRMS.	Completed (September 2014)
Natalizumab	Integrin alpha-4 [32]	NCT05418010	2	**Natalizumab for the Treatment of People With Inflammatory Demyelination Suggestive of Multiple Sclerosis, or Definite Multiple Sclerosis, at First Presentation (AttackMS) (AttackMS).** Evidence suggests that shutting down inflammation using highly effective DMTs early after diagnosis leads to better long-term clinical outcomes. The AttackMS trial will test the effect of starting a highly effective DMT licensed for MS, Tysabri^®^ (Natalizumab 300 mg), within a short time—14 days—after symptom onset.	Recruiting
BGC20-0134	/	NCT01037907	2	**A Study of Orally Administered BGC20-0134 (Structured Lipid) in Patients With Relapsing–Remitting Multiple Sclerosis (RRMS).** To determine the efficacy and safety of an oral drug (BGC20-0134) in patients with RRMS. Specifically, the cumulative number of new gadolinium-enhancing lesions after 24 weeks of treatment with BGC20-0134.	Terminated (December 2011)
EHP-101	Peroxisome proliferator-activated receptor gamma, Cannabinoid receptor 2 [35]	NCT04909502	2	**Evaluation of Safety, Tolerability and Preliminary Efficacy of EHP-101 in Relapsing Forms of Multiple Sclerosis.** The purpose of this trial is to evaluate the safety, tolerability, pharmacokinetics, and preliminary efficacy of EHP-101 in adult subjects with Relapsing Forms of Multiple Sclerosis.	Suspended (April 2024)
Clemastine	Histamine H1 receptor [32]	NCT02521311	2	**Assessment of Clemastine Fumarate as a Remyelinating Agent in Acute Optic Neuritis (ReCOVER).** The main purpose of this study is to assess Clemastine as a remyelinating agent in patients with acute optic neuritis.	Recruiting
Vaginal Estriol	Estrogen receptor alpha [32]	NCT03774407	3	**Vaginal Estriol in Multiple Sclerosis.** Study to evaluate the efficiency of vaginal estriol, as a treatment for urogenital symptoms in female patients with RRMS.	Completed (November 2020)
Clemastine	Histamine H1 receptor [32]	NCT05338450	3	**Clemastine Fumarate as Remyelinating Treatment in Internuclear Ophthalmoparesis and Multiple Sclerosis (RESTORE).** The (long-term) effects of clemastine need to be confirmed in clinical models for MS. Internuclear ophthalmoparesis (INO) may be used as a clinical model for investigating remyelinating therapies by measuring horizontal eye movements with infrared oculography.	Recruiting
Nomegestrol acetate; Estradiol	Progesterone receptor [34]; Estrogen receptor alpha and beta [32]	NCT00127075	3	**POPART’MUS: Prevention of Post-Partum Relapses With Progestin and Estradiol in Multiple Sclerosis.** The POPART’MUS study is a European, multicentre, randomized, placebo-controlled and double-blind clinical trial, which aims to prevent MS relapses related to the post-partum condition, by administering high doses of progestin (nomegestrol acetate), in combination with endometrial protective doses of estradiol.	Unknown
CNM-Au8	NADH [36]	NCT04626921	3	**A Multi-Center, Open-Label Long-Term Extension Study of CNM-Au8 in Patients With Stable Relapsing Multiple Sclerosis (VISIONMS-LTE).** This open-label, long-term extension study is only available to participants who have completed CNMAu8.201 (VISIONARY-MS). The Week 48/End-of-Study Visit for study CNMAu8.201 (VISIONARY-MS) will serve to establish the Baseline for electrophysiological, functional, and morphological vision testing, as well as the neurological and outcome assessments.	Completed (September 2023)
Cladribine	Ribonucleoside-diphosphate reductase large subunit, subunit M2 and M2 B, DNA polymerase alpha catalytic subunit, epsilon catalytic subunit A, epsilon subunit 2, 3 and 4, Purine nucleoside phosphorylase [32]	NCT05902429	4	**Effects of Oral Cladribine on Remyelination and Inflammation in Multiple Sclerosis Patients (CLAREMI).** The present study is based on the hypothesis that improved inflammatory control through cladribine tablets provides a tissue microenvironment more favourable for remyelination of brain lesions in MS.	Active, not recruiting (January 2024)
Alemtuzumab	CAMPATH-1 (CD52) antigen [32]	NCT01395316	4	**Alemtuzumab on Surrogate Markers of Disease Activity and Repair Using Advanced MRI Measures in Subjects with Relapsing–Remitting Multiple Sclerosis.** The MRI study is designed to identify possible mechanisms by which alemtuzumab acts to protect the brain from inflammation and how it may enhance repair through remyelination.	Completed (July 2017)
Rebif	Interferon alpha/beta receptors 1 and 2 [32]	NCT01085318	4	**Rebif Advanced Magnetic Resonance Imaging (MRI) and Immunology Pilot Trial.** The purpose of this trial is to evaluate the effects of Rebif^®^ 44 mcg subcutaneous (sc) three times a week (tiw) on (a) remyelination/demyelination, (b) lesion and brain volume, (c) central nervous system (CNS) iron deposition, and (d) immune status in subjects with RRMS via several MRI techniques.	Completed (March 2012)
Adrenocorticotropin	Adrenocorticotropic hormone receptor [32]	NCT02446886	4	**Adrenocorticotropic Hormone (ACTH) Effects on Myelination in Subjects With MS.** The primary objective of this study is to determine if monthly pulse doses of a three-day course of ACTH (H.P. Acthar^®^) are more effective at recovering myelin at 12 months, as measured by myelin water fraction (MWF), in new MS lesions as compared to one course of treatment.	Terminated (June 2020)

## Data Availability

Not applicable.

## Abbreviations

ADE	Acute Disseminated Encephalomyelitis	
AI	Artificial Intelligence	
aNSCs	activated Neural Stem Cells	
BBB	Blood–Brain Barrier	
Bcas1	Brain-enriched myelin-associated protein 1	
bFGF	basic Fibroblast Growth Factor	
BMPs	Bone Morphogenetic Proteins	
c-Myc	Cellular myelocytomatosis oncogene	
cAMP	cyclic AMP	
CC	Corpus Callosum	
CIS	Clinically Isolated Syndrome	
CNNs	Convolutional Neural Networks	
CNPase	2′,3′-Cyclic-nucleotide 3′-phosphodiesterase	
CNS	Central Nervous System	
CNTF	Ciliary Neurotrophic Factor	
CRISPR	Clustered regularly interspaced palindromic repeats	
CSF	Cerebrospinal Fluid	
CTIP2	COUP-TF-interacting protein 2	
CypD	Cyclophilin D	
CYP51	Sterol 14-demethylase	
DG	Dentate Gyrus	
DLX1	Distal-Less Homeobox 1	
DMTs	Disease-Modifying Therapies	
DSB	Dorsomorphin, SB431542, and BIO	
DTs	Digital Twins	
EAE	Experimental Autoimmune Encephalomyelitis	
EBs	Embryoid Bodies	
EBP	Emopamil Binding Protein	
EGF	Epidermal Growth Factor	
EMX1	Empty Spiracles Homeobox 1	
Enpp8	Ectonucleotide pyrophosphatase/phosphodiesterase 8	
ESCs	Embryonic Stem Cells	
FDA	Food and Drug Administration	
FGF	Fibroblast Growth Factor	
GFP	Green Fluorescent Protein	
Gli1-3	Glioma-associated oncogene family zinc finger proteins	
GPCRs	G protein-coupled receptors	
GPR17	G protein-coupled receptor 17	
GR	Glucocorticoid Receptor	
Hh	Hedgehog	
hiOLs	human iPSCs-derived oligodendrocytes	
IGF1	Insulin-like Growth Factor 1	
iPSCs	induced Pluripotent Stem Cells	
JCV	John Cunningham Virus	
Klf4	Krüppel-Like Factor 4	
LDN	LDN-193189	
LGA	Lamarckian Genetic Algorithm	
LHX6	LIM Homeobox 6	
LINGO-1	Leucine-rich and Immunoglobin-like domain-containing protein 1	
LTP	Long-Term Potentiation	
MACS	Magnetic Activated Cell Sorting	
MAG	Myelin-Associated Glycoprotein	
Mal	Myelin and lymphocyte protein	
MBP	Myelin Basic Protein	
MD	Molecular Dynamics	
ML	Machine Learning	
MRI	Magnetic Resonance Imaging	
MS	Multiple Sclerosis	
MyRF	Myelin Regulatory Factor	
NADH	Nicotinamide adenine dinucleotide reduced form	
NECs	Neuro-Epithelial Cells	
NG2	Neural antigen 2	
NKX2.2	NK2 Homeobox 2	
NMDA	N-methyl-D-aspartate	
NMO	Neuro-Myelitis Optica	
Notch	Neurogenic locus notch homolog protein	
NPCs	Neural Precursor Cells	
NSCs	Neural Stem Cells	
OCT	Optical Coherence Tomography	
Oct3/4	Octamer-binding Transcription factor 3/4	
OLs	myelinating Oligodendrocytes	
Opalin	Oligodendrocytic myelin paranodal and inner loop protein	
OPCs	Oligodendrocyte Precursor Cells	
PAX6	Paired Box 6	
PBMCs	Peripheral Blood Mononuclear Cells	
PDGF	Platelet-Derived Growth Factor	
PDGFRα	Platelet-Derived Growth Factor Receptor alpha	
PLLA	poly(L-lactic acid)	
PLP1	Proteolipid Protein 1	
PM	Purmorphamine	
PMD	Pelizaeus-Merzbacher Disease	
PML	Progressive Multifocal Leukoencephalitis	
pOPCs	parenchymal Oligodendrocyte Precursor Cells	
PPMS	Primary Progressive Multiple Sclerosis	
PS	Polystyrene	
Ptch	Patched	
qNSCs	quiescent Neural Stem Cells	
QSAR	Quantitative Structure–Activity Relationships	
RA	Retinoic Acid	
RMSD	Root–Mean–Square Deviation	
RMSF	Root–Mean–Square Fluctuation	
ROS	Reactive Oxygen Species	
RRMS	Relapsing–Remitting Multiple Sclerosis	
RT-qPCR	Reverse Transcription-quantitative Polymerase Chain Reaction	
S14R	Sterol C-14 reductase	
S1PL	Sphingosine 1-Phosphate receptor Lyase	
SAG	Smoothened agonist	
SDF-1	Stromal cell–derived factor 1	
SGZ	Sub Granular Zone	
Shh	Sonic Hedgehog	
SMAD	Suppressor of Mothers Against Decapentaplegic	
Smo	Smoothened	
SOX1	SRY-box 1	
Sox2	SRY-box 2	
SOX10	SRY-box 10	
SPMS	Secondary Progressive Multiple Sclerosis	
SVZ	Subventricular Zone	
T3	triiodo-L-thyronine	
TBR1	T-box brain Transcription Factor 1	
TBR2	T-box brain Transcription Factor 2	
TGF-β	Transforming Growth Factor-β	
TRPA1	Transient Receptor Potential Ankyrin 1	
